# The Association between Functional Health Patterns and Frailty in Hospitalized Geriatric Patients

**DOI:** 10.3390/geriatrics9020041

**Published:** 2024-03-26

**Authors:** Elisabeta Ioana Hiriscau, Omar Cauli, Valer Donca, Luminita-Aurelia Marinescu, Antonia-Eugenia Macarie, Lucretia Avram, Oana-Gabriela Cancel, Steliana Donca, Elena-Cristina Buzdugan, Dana-Alina Crisan, Constantin Bodolea

**Affiliations:** 1 Nursing Department, Iuliu Hatieganu University of Medicine and Pharmacy, 400083 Cluj-Napoca, Romania; ioanahiriscau@gmail.com; 2 Intensive Care Unit Department, University Clinical Municipal Hospital, 400139 Cluj-Napoca, Romania; cbodolea@gmail.com; 3 Nursing Department, University of Valencia, 46010 Valencia, Spain; 4 Geriatric Department, Iuliu Hatieganu University of Medicine and Pharmacy, 400139 Cluj-Napoca, Romania; valerdonca@gmail.com (V.D.); pascaluminitaaurelia@gmail.com (L.-A.M.); macarieantonia@yahoo.com (A.-E.M.); avram.lucretia9@gmail.com (L.A.); 5 Geriatric Department, University Clinical Municipal Hospital, 400139 Cluj-Napoca, Romania; oana.gabriela29@yahoo.com (O.-G.C.); stelianadonca@gmail.com (S.D.); 6 Internal Medicine Department, Iuliu Hatieganu University of Medicine and Pharmacy, 400139 Cluj-Napoca, Romania; buzelena@yahoo.com (E.-C.B.); crisan.dc@gmail.com (D.-A.C.); 7 Internal Medicine Department, University Clinical Municipal Hospital, 400139 Cluj-Napoca, Romania; 8 Intensive Care Unit Department, Iuliu Hatieganu University of Medicine and Pharmacy, 400139 Cluj-Napoca, Romania

**Keywords:** frailty, functional health pattern, geriatric, nursing, prefrailty

## Abstract

This study investigates the association between the Functional Health Pattern Assessment Screening Tool (FHPAST) and frailty in hospitalized geriatric patients. One hundred and forty patients (mean age 78.2 years, age range 65–90) were screened for frailty using the Frail Scale during hospitalization in the geriatric unit. Among them, 57 patients were identified as prefrail (40.7%), and 83 were identified as frail (59.3%). A comparative analysis between groups in terms of the FHPAST components covering health risk, general well-being, and health promotion was performed. Correlations between FHAPST components, socio-demographic data, frailty criteria, as well as logistic regression to identify variables that better predict frailty were also sought. Frailty was mainly associated with difficulty urinating, limitations in performing activities of daily living and walking, physical discomfort, less positive feelings in controlling one’s own life, lower compliance with recommendations from the healthcare provider, and engagement in seeking healthcare services. Patients with difficulty urinating and walking had a probability of 4.38 times (OR = 4.38, CI 95% [1.20–15.94]), *p* = 0.025) and 65.7 times (OR = 65.7, CI 95% [19.37–223.17], *p <* 0.001) higher of being frail rather than prefrail. The relationship between frailty and prefrailty in hospitalized geriatric patients and components of nursing Functional Health Patterns (FHP) has yet to be explored. This study provides evidence of the most prevalent needs of frail geriatric patients in hospital settings.

## 1. Introduction

Frailty is often associated with aging and is a clinically recognizable state of increased vulnerability due to a decline in reserve and function across different physiological systems (musculoskeletal, neuroendocrine, hematological, immune, and cardiovascular) characterized by a state of chronic low-grade inflammation, with some inflammatory biological markers reflecting this condition (C-reactive protein, interleukin-6, and tumor necrosis factor-alpha) [1,2]. From another perspective, frailty defines a biological syndrome of decreased reserve and resistance to stressors leading to an increased vulnerability to adverse outcomes, such as disability, hospitalization, institutionalization, and mortality [3,4]. Two major conceptualizations are considered relevant for frailty and have been extensively described in the research literature: the first is based on physical functioning (the frailty phenotype), and the second is a model that, in addition to physical frailty, considers the psychological and social domains, as well as chronic diseases, and impairments in performing activities of daily living (ADL) [5,6].

Frailty in older patients is associated with deficits in functional decline, falls, fractures, pressure ulcers, delirium, and a lower quality of life (QoL) [7]. Frailty is related to an increased rate of hospitalization of older individuals in nursing homes or long-term healthcare facilities. It has been reported as the leading cause of death among community-dwelling older people [8]. In hospitalized older patients, frailty predicts poor outcomes upon discharge and increased risk of mortality within 6 months [9,10]. The higher odds of frailty syndrome in hospitalized older patients support the increased risk of adverse events in these patients compared to community-dwelling individuals with similar ages [11,12].

Frailty assessment in hospitalized older individuals is usually performed by clinicians using the Geriatric Comprehensive Assessment (GCA) and rarely or never by nurses. This is partially due to nurses’ perceiving frailty as a state of vulnerability, accompanied by a series of problems that deeply impact the person’s entire functionality, with little focus on a systematic assessment of frailty.

The most frequently used instruments in geriatric units for frailty assessment are the Clinical Frailty Scale (CFS), FRAIL scale, and Reported Edmonton Frail Scale, which each evaluate components such as functional, cognitive, and health status (symptoms, health perception, comorbidities) [6,13,14,15,16]. A recent study aiming to identify the prevalence and frailty-associated factors in hospitalized patients aged 65 and over in internal medicine and surgical clinics reported a frailty prevalence ranging from 46.8 to 57.4%, depending on the tool used to quantify it [13,16,17,18].

Data regarding different frailty screening tools and their applicability in hospital settings within nursing assessment has rarely been reported in studies [19,20]. Even though some countries have developed and validated their frailty screening instruments that have been integrated into the nursing assessment procedure on admission, a holistic frailty assessment framework conducted by nurses has yet to be implemented in hospital settings [21,22]. The biosystems framework continues to be used to assess frailty in many hospitals and health care agencies. The nursing perspective, when approaching frailty in clinical settings, therefore, aims for a systematic assessment framework that provides a holistic picture of the individual’s functioning [23]. Frailty assessment conducted by nurses is needed to fit nursing diagnoses, in order to establish an appropriate care plan and tailor nursing interventions to frail individuals.

The Functional Health Patterns (FHP) model developed by Marjory Gordon is used in clinical nursing and is a comprehensive and systematic method for assessing eleven domains of human functioning. These domains include health perception and management, nutritional-metabolic, elimination, activity exercise, sleep–rest, cognitive-perceptual, self-perception/self-concept, role–relationship, sexuality–reproductive, coping–stress tolerance, and value–belief pattern [24]. Gordon’s FHP assists nurses in identifying health and illness issues related to nursing care, collects data needed to develop care plans, and enables nurses to formulate nursing diagnoses.

The Functional Health Pattern Assessment Screening Tool (FHPAST) is a self-administered instrument developed as an evaluative questionnaire of health, functional, and risk problems. In it, Gordon’s eleven functional health patterns are represented through 57 items with a four-anchor Likert-type response scale, with the items organized into three components: a Health Risk/Threats component, a General Well-Being and Self-Confidence component, and a Health Promotion/Protection Activity component. The scores provide valuable information about the FHPAST components. They can be used in clinical research to establish baseline data about the FHP before and after an experience and to describe responsiveness to nursing interventions [25]. The FHPAST has been tested and found to have satisfactory psychometric properties, which allows researchers to have a complete clinical database quickly. The FHPAST has been used in English-speaking outpatient settings. Projects are underway for its adaptation and refinement in different languages, using appropriate techniques for subsequent use in clinical practice [26]. The questionnaire is simple in form, comprehensible in content, and can be completed in 5–10 min. If the patients cannot complete it independently, the practitioner nurse can read and record the items.

The study’s hypotheses refer to some functional patterns that are more significantly associated with an increase in the severity of frailty (employing comparisons between frail and prefrail patients). We also hypothesized that some FHPAST variables could better predict the presence of frailty in older inpatients in the geriatric ward.

## 2. Materials and Methods

### 2.1. Study Design

This cross-sectional, correlational study aimed to identify the prevalence of frailty in individuals hospitalized in different medical units for one year. A cross-sectional study provides a snapshot of population characteristics, behaviors, or conditions, which allows researchers to explore associations and trends that are especially useful when exploring the relationship between sub-dimensions and distinct measures of common conditions, such as functional patterns and the severity of frailty syndrome. These studies are often used to generate hypotheses, identify trends, and inform public health policies and interventions.

This study is part of the Frail.ro mother-study, an institutional project undertaken at the University Clinical Municipal Hospital, Cluj–Napoca, Romania, between 2016 and 2019. The main objective of the Frail.ro study was to identify the incidence of frailty syndrome in hospitalized populations in different medical units (geriatric, cardiology, surgery, and urology) over the course of one year. Apart from specific instruments used for frailty assessment, we also sought to investigate other frailty-related components, such as nutrition, cognition, psychological performances, social support, and quality of life. The study protocol was approved by the Local Ethics Committee of the University Clinical Municipal Hospital, Cluj–Napoca, Romania (reference protocol no. 5/2017. The study was approved on 20 February 2017).

A multidisciplinary hospital team, including two geriatricians, a clinical psychologist, a geriatric nurse, and three registered nurses, performed the geriatric ward’s frailty assessment between March 2018 and February 2019. The team was trained in a two-hour session before the study started by the project coordinators (the head of the Intensive Care Unit and the clinical psychologist with expertise in the field of nursing for more than 20 years).

The study consisted of two rounds. First, the physicians and nurses gathered data on frailty in the older inpatients in the geriatric ward. Second, the geriatric nurses and other nurses collected data using the FHPAST for the geriatric patients evaluated in the first round. Any discrepancy when collecting FHPAST data from the patient was addressed to the clinical psychologist at the end of the session. The consensus on the problematic item was obtained after analyzing the specific content. The FHPAST data was usually collected on the day following the frailty assessment.

### 2.2. Participants and Sampling

We selected the participants during their hospitalization in the geriatric ward between March 2018 and February 2019. The recruitment involved convenience sampling. We found this method (non-random sampling) appropriate for our study involving hospitalized patients. Their participation in the research was based solely on their agreement. Depending on each patient’s state of health (investigations, treatment, health alteration), we extended the evaluation time from two days to three or four (one potential bias). The second bias was related to the availability of the geriatric nurse (working in shifts), which involved an extra day for finalizing the assessment.

The inclusion criteria were the patients’ agreement to participate and signing the informed consent.

Exclusion criteria were dementia and delirium, chronic inflammatory diseases, the patient being unable to finish the evaluation due to their health, and refusing the comprehensive frailty assessment. One hundred and forty-two patients agreed to participate and signed the informed consent. After performing a frailty assessment, two patients were identified as robust and excluded from the second round of the research. The final sample included 140 patients with complete frailty and FHPAST data.

### 2.3. Variables

The socio–demographic data collected were age and gender, origin, environment, level of education, marital status, lifestyle, self-perceived health, sleep, and income. Polypharmacy (five or more medications taken daily was considered a positive criterion for polypharmacy), body mass index (BMI = weight/height), and comorbidities were collected from medical records. Body mass index (BMI = weight/height^2^) was reported according to the classification proposed by the Mini Nutritional Assessment: 0 = BMI less than 19; 1 = BMI 19 to less than 21; 2 = BMI 21 to less than 23; 3 = BMI 23 or greater. When quantifying the comorbidities, we used the Charlson Comorbidity Index (CCI), a clinically useful tool for validating a patient’s condition and an independent predictor of long-term survival.

We used the FRAIL Scale, a five-question frailty screening tool with a scoring system of Yes = 1/No = 0, with a minimum score of 0 and a maximum of 5 [27]. The areas covered by the FRAIL Scale are: Fatigue—How much of the time during the past 4 weeks did you feel tired?; Resistance—By yourself and not using aids, do you have difficulty walking up ten steps without resting?; Ambulation—By yourself and not using aids, do you have difficulty walking a couple of blocks (e.g., several hundred yards); Illness—Did a doctor ever tell you that you have [illness]?; Loss of weight—How much do you weigh? A score of 3 to 5 characterizes a frail individual, 1 or 2 a prefrail one, and 0 a robust (non-frail) individual.

For Functional Health Pattern Assessment data, we checked each FHPAST item for clarity during the entire translation process from English into Romanian. The FHPAST Health Risk/Threats component included 17 items, the General Well-Being and Self-Confidence component 27 items, and the Health Promotion and Protective Activities component 13 items. All the FHPAST items were considered categorical variables. The total score for the FHPAST components consisted of the positive responses to the items.

### 2.4. Data Collection

This study was conducted in the geriatrics department of a university clinical hospital between March 2018 and February 2019. We collected socio–demographic and relevant clinical data through interviews and by consulting medical records. Frailty was assessed using a short five-question screening tool, while FHPAST data was collected face-to-face.

### 2.5. Ethical Considerations

The study was conducted according to the ethical principles of medical research stated in the Declaration of Helsinki. Written consent was obtained from each patient after they were informed about the purpose of the study, the procedures, and confidentiality regarding the data provided. The study protocol was approved by the Local Ethics Committee of the University Clinical Municipal Hospital, Cluj–Napoca, Romania (reference protocol no. 5/2017).

### 2.6. Data Analysis

Continuous variables were expressed as the mean and standard deviation, and categorical variables were the absolute values with their percentage. We used the chi-squared test to compare the categorical variables. We used the Pearson correlation coefficient for linear correlations between the continuous variables and Spearman’s rank test for the other variables. All the variables of the FHPAST components that correlated with frailty (r > 0.3) were entered into the binary logistic regression to identify those that best predict frailty in hospitalized geriatric patients. Only the statistically significant variables were used in a regression model to predict frailty. Statistical significance was set at a *p* value of less than 0.05. The data analyses were conducted using the IBM SPSS Statistics software package for Windows, version 22.0 (IBM Corp., Armonk, NY, USA).

## 3. Results

The sample included 140 older inpatients, 27 (19%) males and 113 (81%) females. At baseline, 57 (41%) patients were prefrail, and 83 (59%) were frail. The sample baseline characteristics of the prefrail and frail groups and the differences in means between groups in terms of socio–demographic data, polypharmacy, and comorbidities are presented in Table 1. Supplementary Table S1 presents the prevalence of each altered FHPAST component in the prefrail and frail patients.

The differences in means between prefrail and frail hospitalized geriatric patients were related to gender, smoking, alcohol consumption, self-perceived health, and comorbidities. The share of men in the prefrail group was higher than in the frail group. The prefrail older inpatients showed higher levels of alcohol consumption and smoking than the frail patients. In comparison with the prefrail ones, frail inpatients presented a more negative perception of their health and more comorbidities.

The frail patients had lower resistance, more deficits in ambulation, and greater weight loss than the prefrail patients. No differences related to age, environment, education, marital status, sleeping, falls, polypharmacy, and BMI were found between the groups.

Regarding the component of Health Risk/Threat, the frail older inpatients presented more difficulty urinating, feeling unusual physical symptoms when walking, having limitations in performing ADL, and interruptions of daily activities due to pain than the prefrail group. They also experienced more physical discomfort under stress, tension, and the burden of participating in family caregiving activities.

The frail patients expressed fewer positive feelings about controlling their own lives, less capacity to cope with stress, negative feelings regarding their health, and less energy for activities of daily living related to the General Well-Being and Self-Confidence component. They were less open to expressing their feelings and emotions and showed less consistency with their values in life decision-making.

For the component of Health Promotion/Protection Activity, frail patients proved less able to follow recommendations from the healthcare provider, intentionally limit their dietary fat intake, seek immediate attention for changes in their health, or engage in a routine relaxing activity. Compared to the prefrail inpatients, they showed positive behavior regarding the daily consumption of fruits and vegetables.

Positive correlations were found between the Health Risk/Threat component and age (*p* = 0.001, r = 0.35) and smoking (*p* = 0.006, r = 0.30) within the frail group. The General Well-Being and Self-Confidence component was negatively correlated with age in both the prefrail (*p* = 0.03, r = −0.29) and frail older patients (*p* = 0.001, r = −0.42) and positively correlated with self-perceived health (*p* = 0.006, r = 0.36), and sleeping (*p* = 0.031, r = 0.29) in the prefrail patients, respectively, and with sleeping in the frail patients (*p* = 0.012, r = 0.27). The Health Promotion/Protective Activity component was negatively correlated with age in the prefrail older patients (*p* = 0.049, r = −0.26) and positively with self-perceived health (*p* = 0.001, r = 0.51) and sleeping (*p* = 0.047, r = 0.27); in the frail patients, it was negatively correlated with age (*p* = 0.001, r = −0.36), and positively with self-perceived health (*p* = 0.004, r = 0.31). Correlations between the FHPAST and gender, environment, level of education, marital status, and alcohol consumption were not statistically significant in either the prefrail or the frail group. Only correlations above 0.03 were considered relevant for logistic regression.

**Figure 1:**
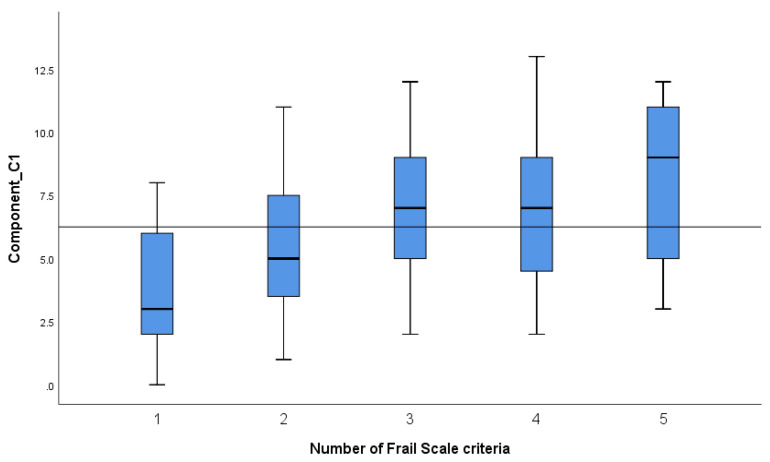
The mean scores for the Frail Scale criteria for the Health Risk/Threat (Component C1).

**Figure 2:**
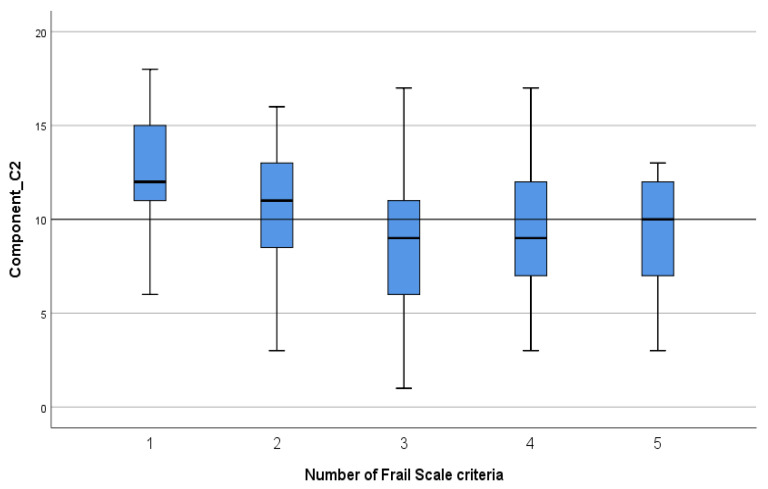
The mean scores for the Frail Scale criteria for General Well-Being and Self-Confidence (Component C2).

**Figure 3:**
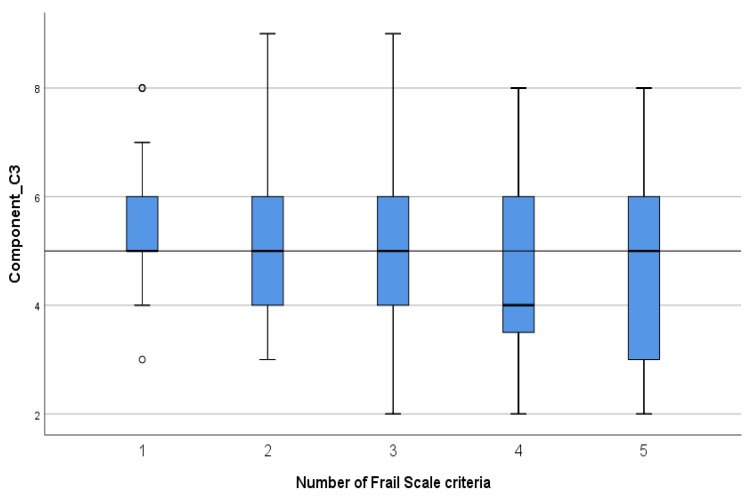
The mean scores for the Frail Scale criteria for Health Promotion/Protection Activity (C3).

Statistically significant correlations were found between the Frail Scale criteria and FHPAST components as follows: a positive correlation (*p* < 0.001, r = 0.44) between frailty criteria and the Health Risk/Threat component illustrates that an increase in the number of criteria is associated with increased health risks (Figure 1); a negative correlation (*p* < 0.001, r = −0.33) between frailty criteria and the General Well-being and Self-Confidence component shows that an increase in the number of criteria is associated with a decline in general well-being and Self-Confidence (Figure 2); and a negative correlation between frailty criteria and the Health Promotion/Protective activity component (*p* = 0.009, r = −0.22) shows that an increase in the number of criteria is associated with a decline in health–protective activities (Figure 3).

Table 2 illustrates the logistic regression results for frailty as the outcome variable.

The predictor variables were selected after searching the correlations > 0.3 between frailty and the items in each FHPAST component. In order to build the model that predicts the outcome variable (frailty), three predictors, C1.3 (I have difficulty urinating), C1.4 (I feel unusual physical symptoms when walking), and C3.3 (I intentionally limit my dietary fat intake), were entered in the binary logistic regression. The overall percentage explaining the model is 89.3%. C1.3 and C1.4 were statistically significant in predicting frailty in hospitalized geriatric patients.

Patients who did not fulfill criterion C1.3 (I have difficulty urinating) had a significantly lower probability of being frail (OR = 0.228, CI 95% 0.063–0.830, *p* = 0.025). Patients who did not fulfill criterion C1.4 (I feel unusual physical symptoms when walking) had a significantly lower probability of being frail (OR = 0.015, CI 95% 0.004–0.052, *p <* 0.001). Item C3.3 (I intentionally limit my dietary fat intake) did not reach statistical significance. Odds ratios calculated by dividing one by the values of Exp(B) as the presence of the item in logistic regression showed a probability 4.38 times higher of being frail rather than prefrail for the patients who had “difficulty urinating”, and a probability 65.7 times higher of being frail rather than prefrail for those who had “unusual physical symptoms when walking”.

## 4. Discussion

Originally developed as an instrument for nurses to screen functional health within the FHP framework in clinical practice, FHPAST has also proved reliable and valid for research use. The development of tools to screen FHP was a way to streamline the assessment process in diagnosing patients’ problems, risk of problems, and readiness for health, as well as designing outcomes responsive to nursing interventions. The FHP framework provides a structure for uncovering and describing human responses at a complex, holistic level while integrating biophysiological and behavioral functioning [24]. The FHPAST was developed to establish a reliable and valid instrument to screen the FHPs across populations and settings. The FHPAST item level scores provide valuable information about specific aspects of the three components of functional health [25]. The FHPAST could be further used in research to establish baseline data about the functional health patterns in older individuals [28] before and following a stressful event or to analyze the effects of therapeutic intervention [29,30,31]. A fundamental approach in the field of nursing and healthcare is Gordon’s Functional Patterns, which provide an invaluable tool for carrying out a comprehensive assessment of the patient, focusing on different aspects of their life and health [32]. Knowing and understanding these functional patterns provides a more complete view of the health of older individuals and their needs. Early identification of worsening frailty syndrome is associated with the alteration of some of FHP and can help nurses begin preventive care in combination with nursing care and additional tailored geriatric care [33,34]. Multidimensional interventions in the physical, nutritional, psychological, and social domains are effective and can prevent negative health outcomes associated with frailty in hospitalized older individuals [35]. Multi-domain (e.g., related to physical, mental health, and psychological, social, environmental, and economic factors) frailty screening, including FHP evaluation, is crucial for nurses to implement a proper care plan and is necessary to support interdisciplinary collaboration to support older patients effectively [36].

Like FHP, frailty is a multidimensional and dynamic process that can be reversed through specific interventions and health strategies, especially in the early stages of frailty [37]. Previous studies have shown that the reversal rate from pre-frailty to health is 23.3% [38]. Timely interventions are needed in both prefrail and frail geriatric patients to prevent the occurrence and development of frailty or to limit the deficits already occurring that impact the individual’s autonomy after hospitalization [4,13]. If appropriate frailty interventions are not promptly implemented, the frailty may progress and lead to partial or total loss of functional autonomy (physical, psychological, social), thereby increasing the risk of prolonged hospital stays, functional decline on discharge, an increased medical and economic burden of social and family care, and a high mortality rate in the medium- and long-term [39,40].

Nurses play a central role in detecting frailty in hospitalized older people using validated screening and assessment tools. Since frailty is affected by multiple factors, it is highly appropriate to use a holistic nursing approach to frailty assessment in hospital settings and to plan and implement multidimensional interventions that target multiple contributing factors. Nurses need training in performing frailty screening in geriatric acute care settings, using screening tools as described above, and understanding how the frailty score will be processed for decision-making in developing and planning the care [16].

Using FHPAST in a geriatric care setting enables nurses to formulate nursing diagnosis that covers deficits in ADL, nutrition, elimination, symptom control, physical discomfort, and psychological distress related to health risks, issues related to well-being such as coping with stress, roles, and relationships, self-management, values, and beliefs and health protection activities. Besides the physical characteristics of frailty, this construct also considers bio-psycho-social factors. Cognitive and social frailty is also linked to negative consequences in older adults, which are covered by the FHPAST components of Self-Confidence and Health Promotion/Protection Activity in terms of abilities related to problem-solving, communication with others, coping with stress, promoting social behaviors and social activities, and self-management abilities [41,42]. From the nursing perspective, these are crucial in frailty assessment. The biomedical framework does not cover them, but they are the mainstay of nurses’ competencies.

For the Health Risk/Threats component, an increase in the number of frailty criteria means more health risks/threats, especially in the transition from pre-frailty (1 or 2 criteria) to frailty (3 criteria) when older people are more likely to develop health deficits that need to be compensated during hospitalization. Although frailty is advancing, the transition from 3 to 4 criteria is less severe for health risks, with a significant increase seen in the transition from 4 to 5 criteria.

For the General Well-Being and Self-Confidence component, an increase in frailty criteria is related to poorer general well-being and Self-Confidence in frail older inpatients. Prefrail older inpatients seem to experience a significant decline in their health status when transitioning from 1 to 2 criteria, gradually descending until frailty (3 criteria) is reached. From this critical point, frail elderly people are more likely to have only slight variations in their health. Targeted nursing interventions in the transition from pre-frailty to frailty might have benefits in terms of conserving health resources and delaying the onset of frailty.

For the Health Promotion/Protection Activity component, increasing the number of frailty criteria is related to a decline in health promotion and protection activities. The transition in the pre-frailty area (from 1 to 2 criteria) and from pre-frailty to frailty stage 3 or 4 marks a constant decline in health–protective activities, but less so in the transition from 4 to 5 criteria.

Two main predictors for frailty were identified in our study: the presence of unusual physical symptoms when walking and difficulty urinating. The odds of “I feel unusual physical symptoms when walking” were 65.7 times higher in frail geriatric inpatients than in prefrail inpatients. “Feeling unusual physical symptoms when walking” was mainly expressed by slowness in geriatric inpatients. A walking speed of less than one m/s was found to predict frailty and was associated with disability, hospitalization, and decreased survival. In older inpatients, the average walking speed reported in studies was 0.52–0.58 m/s, depending on the tools used for frailty assessment [43,44,45]. Accordingly, any change in walking during hospitalization might be considered an alarm signal for advancing frailty conditions in geriatric inpatients. The odds of “I have difficulty urinating” were 4.38 times higher in frail geriatric inpatients than in their prefrail counterparts. The relationship between frailty and severity of lower urinary tract symptoms (LUTS) was examined in many studies, and severe LUTS was associated with exhaustion and low levels of physical activity [46]. Considering these symptoms within the assessment of geriatric inpatients may help clinical nurses to rapidly identify those in a prefrail condition who are at risk of becoming frail during hospitalization. By being aware of any changes in these behaviors, clinical nurses in the geriatric ward could contribute to preventing advancing frailty through appropriate and tailored interventions in geriatric inpatients.

### Limitations

Some limitations of the present study warrant consideration. First, the patients recruited for our study were classified as prefrail and frail. Comparing robust, prefrail, and frail older inpatients was impossible under these research conditions. The results are, therefore, limited to the geriatric prefrail and frail inpatients. The robust geriatric inpatients and those transitioning from robustness to pre-frailty have not been taken into consideration.

Second, some FHPAST items proved to be less specific for characterizing the behaviors of the older population or less present in the social culture of the older individuals interviewed. Data regarding the use of recreational drugs, feelings of guilt associated with drinking alcohol, self-image, sexuality, wearing a seat belt, and use of sunscreen could not be appropriately quantified based on the responses provided by the patients and were considered missing values in the study.

## 5. Conclusions

Frailty assessments by nurses in hospital settings should be performed systematically, permitting data collection from a holistic perspective. FHPAST is an instrument that can be used as a framework for assessing frailty in hospitalized geriatric patients, which covers health risks, general well-being, and protective activities. Despite its promising results, FHPAST should not be used in clinical settings as an independent assessment instrument but in conjunction with characteristics and tools targeting specific deficits and needs. Further research is needed to prove the clinical value of FHPAST in frailty assessment in other medical fields, such as cardiac surgery or general surgery. This study has laid the foundations for further analysis of new criteria (functional health patterns) for design interventions for frailty or preventing the progression from pre-frailty to frailty in geriatric patients. Future longitudinal studies clearly need to evaluate a possible relationship between altered functional patterns and the evolution of frailty syndrome severity over time because a cross-sectional study cannot infer a causal relationship.

## Figures and Tables

**Table 1:** Characteristics of the sample included in the analysis. The quantitative variables are expressed as mean (M) and standard deviation (SD), and the qualitative variables are expressed as percentages.

Variables		Prefrail Group (N = 57/140)	Frail Group (N = 83/140)	*p* Value
		Mean (SD) or n (%)	Mean (SD) or n (%)	
**Age (years)**		77.8 (6.3)	78.5 (6.4)	0.713
**Gender**	**Males**	16 (28)	11 (13)	***<0.001***
**Origin**	**Urban**	24 (42)	35 (42)	0.258
	**Rural**	33 (58)	48 (58)	
**Education level**	**Elementary**	37 (65)	55 (66)	0.559
	**Secondary**	15 (26)	21 (25)	
	**University (and higher)**	5 (9)	7 (9)	
**Marital status**	**Married**	20 (35)	26 (31)	0.704
	**Single/Widow**	37 (65)	57 (69)	
**Smoking**	**Active Smoker**	13 (23)	10 (12)	***0.04***
**Alcohol** **consumption ^a^**	**≤7 (14) u/week**	47 (82)	82 (99)	***0.003***
	**≥7 (14) u/week**	10 (18)	1 (1)	
**Self-perceived health**	**Poor**	21 (37)	49 (59)	0.082
**Sleeping**	**Poor**	36 (62)	58 (70)	0.581
**Falls** **(within the last year)**	**0 = no falls**	32 (56)	33 (40)	0.058
	**1 = more than one fall**	25 (44)	50 (60)	
**Polypharmacy** **(medications taken daily)**	**<5 medications**	24 (42)	22 (27)	0.06
	**>5 medications**	33 (58)	61 (73)	
**BMI ^b^**	**BMI < 19**	4 (7)	3 (4)	0.437
	**BMI 19–21**	1 (2)	12 (14)	
	**BMI 21–23**	11 (19)	13 (16)	
	**BMI > 23**	41 (72)	55 (66)	
**CCI ^c^**		6.8 (1.7)	8.0 (2.2)	***<0.001***
**Frail scale**	**Fatigue**	48 (80)	73 (88)	0.241
	**Resistance**	5 (8)	65 (79)	***<0.001***
	**Ambulation**	8 (13)	69 (83)	***<0.001***
	**Illnesses**	31 (52)	57 (69)	0.163
	**Loss of weight**	15 (25)	50 (60)	***<0.001***

Notes: Variables in bold are significant at *p* < 0.05. ^a^ Alcohol consumption (1 unit = 250 mL beer or 75 mL wine or 25 mL brandy); ^b^ Body Mass Index (kg/m^2^); ^c^ CCI (Charlson Comorbidity Index).

**Table 2:** Logistic regression results.

FHPAST Variables	B	Sig.	Exp (B)	95% C.I. for EXP (B)	
				Lower	Upper
C1.3 (I have difficulty urinating)	−1.48	***0.025***	0.23	0.06	0.83
C1.4 (I feel unusual physical symptoms when walking)	−4.19	***<0.001***	0.02	0.00	0.05
C3.3 (I intentionally limit my dietary fat intake)	0.97	0.098	2.64	0.84	8.32

Notes: Variables in bold are significant at *p* < 0.05.

## Data Availability

Research data will be shared on reasonable request.

## Supplementary Materials

The following supporting information can be downloaded at: https://www.mdpi.com/article/10.3390/geriatrics9020041/s1, Table S1: Characteristics of the sample related to FHPAST. Qualitative variables are expressed as percentages.
