# Supplementary material for: The Association between Functional Health Patterns and Frailty in Hospitalized Geriatric Patients
Source: Geriatrics (Basel). 2024 Mar 26;9(2):41. doi: 10.3390/geriatrics9020041 (PMC11050315; doi:10.3390/geriatrics9020041)
Supplement: Supplementary file 1 [file geriatrics-09-00041-s001.zip › geriatrics-2892954-supplementary.pdf]

Supplementary Table S1. Characteristics of the sample related to FHPAST. Qualitative variables are expressed as percentages.

FHPAST components	Variables	Prefrail group (N= 57/140)	Frail group (N= 83/140)	*P value*
		n (%)	n (%)	
FHPAST – C1[a] Health Risk/Threat	C1.1 I fear for my safety	31 (54)	42 (51)	0.436
	C1.2 I feel at risk for physical harm	22 (39)	23 (28)	0.185
	C1.3 I have difficulty urinating	12 (21)	42 (51)	<0.001
	C1.4 I feel unusual physical symptoms with walking	8 (14)	78 (94)	<0.001
	C1.5 I use recreational drugs	0	0	0
	C1.6 I have problems with bowel elimination	9 (16)	20 (24)	0.223
	C1.7 My physical abilities limit my activities of daily living	20 (35)	62 (75)	<0.001
	C1.8 I experience pain that interrupts my daily activities	18 (32)	43 (52)	0.016
	C1.9 When I drink alcohol, wine, or beer, I feel guilty	0	0	0
	C1.10 I have difficulty controlling my anger	8 (14)	8 (10)	0.440
	C1.11 I have family problems that I find difficult to handle	17 (30)	22 (27)	0.672
	C1.12 I experience physical discomfort when I am under stress	22 (39)	55 (66)	<0.001
	C1.13 I smoke cigarettes	4 (7)	5 (6)	0.818
	C1.14 It is a burden to participate in family caretaking activities	15 (26)	50 (60)	<0.001
	C1.15 I have difficulty with my vision	42 (74)	69 (83)	0.192
	C1.16 I feel stress, tension, or pressure	28 (49)	53 (64)	0.04
	C1.17 I worry a lot	15 (26)	30 (36)	0.217

FHPAST – C2[b] General Well-Being and Self Confidence	C2.1 I feel good about myself	33 (58)	40 (48)	0.142
	C2.2 I feel in control of my life	18 (32)	8 (10)	0.008
	C2.3 I feel good about the decisions I make	15 (26)	19 (23)	0.950
	C2.4 I like the way I look	0	0	0
	C2.5 I am happy with my life	50 (88)	54 (65)	0.001
	C2.6 I am hopeful about the future	40 (70)	45 (54)	0.119
	C2.7 I am satisfied with my problem-solving ability	42 (74)	57 (69)	0.795
	C2.8 I am able to cope with stresses in my life	45 (79)	51 (64)	0.008
	C2.9 I consider myself to be healthy	45 (79)	48 (58)	0.059
	C2.10 I am able to adjust to changes in my life	46 (81)	62 (75)	0.674
	C2.11 I have enough energy for activities of daily living	26 (46)	13 (16)	0.003
	C2.12 I am satisfied with my social life	3 (5)	3 (4)	0.650
	C2.13 I am in excellent health	3 (5)	3 (4)	0.650
	C2.14 I heal easily	22 (37)	40 (48)	0.263
	C2.15 I am able to learn new information easily	25 (44)	25 (30)	0.167
	C2.16 I feel comfortable with the role I play in my family	39 (68)	59 (71)	0.484
	C2.17 I fall asleep without a problem	21 (37)	25 (30)	0.241
	C2.18 I feel I can easily communicate with others	19 (33)	20 (24)	0.243
	C2.19 I can concentrate for a long period of time	12 (21)	11 (13)	0.461
	C2.20 I feel rested when I awake	4 (7)	4 (5)	0.598
	C2.21 I feel comfortable with my weight	19 (33)	16 (19)	0.150

	C2.22 The choices I make about my life are consistent with my values	46 (81)	53 (64)	0.069
	C2.23 I am satisfied with what I do for work	0	0	0
	C2.24 I feel comfortable expressing my feelings and emotions	38 (67)	38 (46)	0.005
	C2.25 I have someone I can talk to when I need help/support	30 (53)	48 (58)	0.370
	C2.26 I am comfortable with my sexuality	0	0	0
	C2.27 I am able to hear clearly	26 (46)	41 (49)	0.663
FHPAST – C3[c] Health Promotion/ Protective Activities	C3.1 I have an annual health examination	21 (37)	29 (35)	0.819
	C3.2 I am able to follow recommendations from my healthcare provider	36 (63)	36 (43)	0.021
	C3.3 I intentionally limit my dietary fat intake	42 (74)	29 (35)	<0.001
	C3.4 I eat five to six servings of fruits and vegetables daily	18 (32)	46 (55)	0.001
	C3.5 I seek immediate attention for changes in my health	30 (53)	20 (24)	0.002
	C3.6 I wear a seat belt	0	0	0
	C3.7 My health is important to me	46 (83)	75 (90)	0.193
	C3.8 Religious/ spiritual practices give meaning to my life	36 (63)	55 (66)	0.707
	C3.9 I avoid the sun or use sunscreen	0	0	0
	C3.10 I drink six to eight glasses of water daily	4 (7)	17 (21)	0.071
	C3.11 I can make changes in my lifestyle to improve my health	15 (26)	19 (23)	0.399
	C3.12 I do aerobic exercise for 20 min 23 or more times a week	12 (21)	15 (18)	0.396

	C3.13 I have a usual routine that I perform to help me relax	44 (77)	51 (61)	0.016

Notes: Variables in bold are significant at $p < 0.05$. FHPAST - C1[a] (Functional Health Patterns Assessment Screening Tool – component 1); FHPAST - C2[b] (Functional Health Patterns Assessment Screening Tool – component 2); FHPAST - C3[c] (Functional Health Patterns Assessment Screening Tool – component 3).
